# Association of Grit Scores With Treatment Adherence and Biomarkers in Patients With Type 2 Diabetes

**DOI:** 10.1001/jamanetworkopen.2019.11476

**Published:** 2019-09-13

**Authors:** Pablo A. Peña, Iván Pérez-Díaz, Ana K. Pulido-Ayala, Hillary K. Osorio-Landa, Juan M. López-Navarro, Angela L. Duckworth, Anupam B. Jena

**Affiliations:** 1 Kenneth C. Griffin Department of Economics, University of Chicago, Chicago, Illinois; 2 National Institute of Medical Science and Nutrition Salvador Zubirán, Mexico City, Mexico; 3 Tecnológico de Monterrey, Mexico City, Mexico; 4 Department of Psychology, University of Pennsylvania, Philadelphia; 5 Department of Health Care Policy, Harvard Medical School, Boston, Massachusetts

## Abstract

This cohort study analyzes the association of grit scores with treatment adherence, as measured with a survey and biomarker levels, among patients with type 2 diabetes.

## Introduction

One in 12 adults worldwide has diabetes.^1^ Medical treatment can reduce diabetes-related complications, but adherence to treatment can be difficult.^2^ The reasons for nonadherence are multifactorial. Social scientists have found that character skills—sometimes referred to as socioemotional, noncognitive, or soft skills—are an important factor in whether people succeed in long-term challenges. For instance, the concept of grit, which is defined as “perseverance and passion for long-term goals,”^3^ is associated with success in challenging domains and is independent of intelligence. This cohort study explores whether scores on the grit scale are associated with treatment adherence and biomarker levels in patients with type 2 diabetes.

## Methods

The ethics committee of the National Institute of Medical Science and Nutrition Salvador Zubirán approved the study. The study participants provided written informed consent. The reporting of this study conforms to the Strengthening the Reporting of Observational Studies in Epidemiology (STROBE) reporting guideline.^4^

Between October 2017 and November 2018, surveys were conducted among patients with type 2 diabetes at the hospital of the National Institute of Medical Science and Nutrition Salvador Zubirán immediately before their scheduled medical consultation. The survey included 4 questions on self-reported adherence to treatment, diet, and exercise, as well as the Short Grit Scale (Grit-S), which is computed as the mean of 8 items in which patients rate their perseverance of effort and consistency of interest on a 5-point Likert scale.^5^ Unlike patient-engagement metrics, the Grit-S does not include questions about a patient’s knowledge of the disease or treatments and, therefore, is a priori independent of medical outcomes. Patient socioeconomic information and laboratory data from a few days before the scheduled medical consultation and survey date were obtained from hospital records.

Multivariable linear regression analysis of adherence, biomarkers, and Grit-S scores was performed both with and without adjustment for covariates, which included 7 categories of socioeconomic status (determined and coded by hospital social workers on the basis of patient information, such as household income, dwelling characteristics, and occupation of the primary income earner) and cubic polynomials for years of schooling and number of years with diabetes. Analysis was performed using Stata statistical software version 15.0 (StataCorp), and the 95% confidence interval around estimates reflects 0.025 in each tail; 2-tailed *P* ≤ .05 (*t* test) was considered statistically significant.

## Results

A total of 442 patients (275 [62%] female; mean [SD] age, 64 [12] years; age range, 24-93 years) were surveyed in person. The response rate was 100%. The patients had a mean (SD) of 10 (5) years of schooling (range, 0-26 years) and a mean (SD) of 17 (10) years with type 2 diabetes (range, 0-47 years). The percentage of patients who adhered to treatment ranged between 48.5% and 72.3%, depending on the measure of adherence (Table 1). Grit was associated with greater adherence in all measures. An increase of 1 SD in the Grit-S score was associated with an increase in adherence of 6.1% to 12.3%, depending on the measure analyzed (Table 1). Results were similar with multivariable adjustment.

**Table 1.** Grit and Treatment Adherence

Measure of Treatment Adherence^a^	Patients, No.	Patients Adherent, %	Change in Adherence Associated With a 1-SD Increase in the Short Grit Scale Score, % (95% CI)	
			Univariate	After Multivariate Adjustment^b^
Took medication as prescribed for each day in the last mo	439	66.5	12.3 (7.9-16.6)	12.4 (7.7-17.0)
Did not experience a medication interruption in the last mo	440	72.3	6.1 (1.9-10.3)	6.4 (1.9-10.8)
Exercised ≥3 d in last wk	441	51.7	8.0 (3.3-12.7)	8.0 (3.0-12.9)
Followed recommended diet >60% of the time	441	48.5	8.3 (3.6-13.0)	7.0 (2.1-12.0)

^a^ Estimates were computed with separate regressions. Sample sizes range between 407 and 431 patients.

^b^ Covariates included an indicator variable for sex, cubic polynomials in years of schooling and number of years with diabetes, and categorical variables for several measures of socioeconomic status determined by hospital social workers according to household income, dwelling characteristics, and occupation of the primary income earner.

Mean (SD) laboratory values were as follows: triglycerides, 174.9 (132.8) mg/dL (to convert to millimoles per liter, multiply by 0.0113); total cholesterol, 175.0 (47.8) mg/dL (to convert to millimoles per liter, multiply by 0.0259); glycated hemoglobin, 8.17% (1.99%) (to convert to proportion of total hemoglobin, multiply by 0.01); and serum glucose, 145.1 (60.1) mg/dL (to convert to millimoles per liter, multiply by 0.0555) (Table 2). Grit was associated with lower levels of each measure. An increase of 1 SD in the Grit-S score was associated with lower triglyceride (change, −17.8 mg/dL; 95% CI, −30.5 to −5.1 mg/dL), cholesterol (change, −6.3 mg/dL; 95% CI, −10.9 to −1.7 mg/dL), glycated hemoglobin (change, −0.17%, 95% CI, −0.36% to 0.02%), and glucose (change, −5.1 mg/dL, 95% CI, −10.8 to 0.7 mg/dL) levels. Results were similar with multivariable adjustment.

**Table 2.** Grit and Measures of Glycemic and Cholesterol Control

Outcome^a^	Mean (SD)	Patients, No.	Change in Outcome Associated With a 1-SD Increase in the Short Grit Scale Score (95% CI)	
			Univariate	After Multivariate Adjustment^b^
Triglycerides, mg/dL	174.9 (132.8)	425	−17.8 (−30.5 to −5.1)	−18.5 (−32.2 to −4.8)
Cholesterol, mg/dL	175.0 (47.8)	425	−6.3 (−10.9 to −1.7)	−5.2 (−10.0 to −0.4)
Glycated hemoglobin, %	8.17 (1.99)	425	−0.17 (−0.36 to 0.02)	−0.07 (−0.26 to 0.13)
Glucose, mg/dL	145.1 (60.1)	433	−5.1 (−10.8 to 0.7)	−6.3 (−12.3 to −0.2)

SI conversion factors: To convert triclycerides to millimoles per liter, multiply by 0.0113; cholesterol to millimoles per liter, multiply by 0.0259; glycated hemoglobin to proportion of total hemoglobin, multiply by 0.01; and glucose to millimoles per liter, multiply by 0.0555.

^a^ Estimates were computed with separate regressions. Sample sizes range between 392 and 422 patients.

^b^ Covariates included an indicator variable for sex, cubic polynomials for years of schooling and number of years with diabetes, and categorical variables for several measures of socioeconomic status determined by hospital social workers according to household income, dwelling characteristics, and occupation of the primary income earner.

## Discussion

To our knowledge, this study is the first to analyze the association between patient Grit-S score, treatment adherence, and measures of disease control. Among patients with type 2 diabetes, grit was associated with greater treatment adherence and better glycemic and cholesterol control. Because character skill scales used by social scientists can be routinely measured among patients as they start treatment, these findings suggest that programs aimed at increasing treatment adherence may optimally focus on patients who, on the basis of the scores in those scales, are likely to display low adherence. These findings also suggest the importance of soft skills in overcoming long-term health challenges, such as managing diabetes. Study limitations include the analysis of observational data, which precludes a causal interpretation of the association between grit and treatment adherence, a focus on patients with type 2 diabetes, and analyses performed in a single center.
